# Opposing inflammatory biomarker responses to sleep disruption in cancer patients before and during oncological therapy

**DOI:** 10.3389/fnins.2022.945784

**Published:** 2022-09-21

**Authors:** Delmy Oliva, Bengt-Åke Andersson, Freddi Lewin, Lasse D. Jensen

**Affiliations:** ^1^Department of Oncology, Ryhov County Hospital, Jönköping, Sweden; ^2^Department of Biomedical and Clinical Sciences, Linköping University, Linköping, Sweden; ^3^Department of Natural Science and Biomedicine, School of Welfare, Jönköping University, Jönköping, Sweden; ^4^Division of Diagnostics and Specialist Medicine, Department of Health, Medicine and Caring Sciences, Linköping University, Linköping, Sweden

**Keywords:** cancer, sleep, inflammation, oncological therapy, biomarkers

## Abstract

**Background:**

Sleep disruption is known to be highly prevalent in cancer patients, aggravated during oncological treatment and closely associated with reduced quality of life, therapeutic outcome and survival. Inflammatory factors are associated with sleep disruption in healthy individuals and cancer patients, but heterogeneity and robustness of inflammatory factors associated with sleep disruption and how these are affected by oncological therapy remain poorly understood. Furthermore, due to the complex crosstalk between sleep-, and therapy-associated factors, including inflammatory factors, there are currently no established biomarkers for predicting sleep disruption in patients undergoing oncological therapy.

**Methods:**

We performed a broad screen of circulating biomarkers with immune-modulating or endocrine functions and coupled these to self-reported sleep quality using the Medical Outcomes Study (MOS) sleep scale. Ninety cancer patients with gastrointestinal, urothelial, breast, brain and tonsillar cancers, aged between 32 and 86 years, and scheduled for adjuvant or palliative oncological therapy were included. Of these, 71 patients were evaluable. Data was collected immediately before and again 3 months after onset of oncological therapy.

**Results:**

Seventeen among a total of 45 investigated plasma proteins were found to be suppressed in cancer patients exhibiting sleep disruption prior to treatment onset, but this association was lost following the first treatment cycle. Patients whose sleep quality was reduced during the treatment period exhibited significantly increased plasma levels of six pro-inflammatory biomarkers (IL-2, IL-6, IL-12, TNF-a, IFN-g, and GM-CSF) 3 months after the start of treatment, whereas biomarkers with anti-inflammatory, growth factor, immune-modulatory, or chemokine functions were unchanged.

**Conclusion:**

Our work suggests that biomarkers of sleep quality are not valid for cancer patients undergoing oncological therapy if analyzed only at a single timepoint. On the other hand, therapy-associated increases in circulating inflammatory biomarkers are closely coupled to reduced sleep quality in cancer patients. These findings indicate a need for testing of inflammatory and other biomarkers as well as sleep quality at multiple times during the patient treatment and care process.

## Introduction

Cancer patients are at higher risk of developing sleep disruption pathologies such as insomnia and excessive daytime sleepiness (Fiorentino and Ancoli-Israel, 2007; Savard et al., 2015). It is estimated that sleep disruption is 3–5 times more prevalent among cancer patients compared to the general population, exceeding 50% of the entire patient group in some studies (Divani et al., 2022). As sleep disruption in cancer patients is closely linked to reduced quality of life and survival (Innominato et al., 2012), timely diagnosis and treatment is of critical importance to extend high quality life expectancy in this patient group. A clear example of the impact sleep disruption may have on cancer patients was reported by Innominato et al. (2012) who discovered that survival was extended by almost 8 months in colorectal cancer patients with robust daily (circadian) sleep patterns (49% of the population) compared to patients with disrupted sleep patterns (Innominato et al., 2012). As such, being able to rapidly and routinely identify patients at risk of suffering from poor sleep quality may have enormous prognostic value in oncological practice.

### Clinical diagnosis of sleep disruption in cancer patients

There are currently no instruments implemented into routine clinical practise for diagnosing or quantifying the quality of sleep or extent of sleep disruption in cancer patients. Oncology guidelines such as the Pan-Canadian guidelines, however, raise the importance of assessing, preventing and treating sleep disruption in adult cancer patients (Howell et al., 2013). These guidelines propose an algorithm that starts with the diagnosis of a “sleep problem” based on the patient responding “yes” to a question if he/she suffers from “sleep problems,” followed by asking whether this has persisted for more than three nights and is influencing the patients daytime function (Howell et al., 2013). While this is a somewhat crude assessment of sleep disruption, these guidelines represent the first attempt to include this important issue in routine cancer care. Within clinical research, however, numerous questionnaires and other instruments have been developed to investigate sleep disruption in cancer patients [recently reviewed by Jensen et al. (2021)]. The most commonly used is the extensive 19-item Pittsburgh Sleep Quality Index (Divani et al., 2022), which however is time-consuming to both fill and analyze. A shorter and more intuitive form is the 12-item Medical Outcomes Study (MOS) Sleep Scale which, in spite of the condensed format, evaluates multiple aspects of sleep quality including sleep duration, somnolence, and sleep-related daily function (Bower et al., 2000). The methodology applied to analyze the responses to the MOS Sleep Scale has, however, differed within the research community, and has in the past only included fragments of the scale. Currently an intuitive and unified readout from the MOS scale that take all the answers into account in a clinically meaningful manner, is lacking. The development of such a readout would greatly facilitate the use of this instrument, and indeed the routine and standardized evaluation of sleep quality in cancer patients in general.

### Sleep and inflammation

Cytokines and chemokines are small proteins that control local and systemic immune and inflammatory reactions in the organism and are thought to play a significant role in sleep (Irwin et al., 2008: Kapsimalis et al., 2008). Pro-inflammatory cytokines such as tumor necrosis factor (TNF)-a, interferon (IFN)-gamma, interleukin (IL)-2, 6 and 12, and granulocyte-macrophage colony stimulating factor (GM-CSF) are known to induce daytime sleepiness (Mantovani et al., 2008). On the other hand, sleep disruption is also linked to augmented inflammatory processes through their cytokine- and chemokine-mediated control mechanisms (Irwin et al., 2013; Wright et al., 2015). As such, pro-inflammatory cytokines are often found at elevated levels in both cancer patients and people suffering from poor sleep [reviewed in Jensen et al. (2021)], and are associated with nausea, depression and a generalized feeling of illness (Vgontzas et al., 2007; Kapsimalis et al., 2008; Vumma et al., 2017). In addition to inflammatory cytokines and chemokines, C-reactive protein (CRP) is a commonly used biomarker of inflammation (Sproston and Ashworth, 2018). CRP is commonly used to monitor potential infection during oncological treatment (Howren et al., 2009; Bribriesco, 2020), but has also been suggested as a biomarker of sleep disruption (Jensen et al., 2021). The associations between deregulated circulating levels of inflammatory biomarkers including CRP and sleep disruption are however still poorly understood, and to what extent these are influenced by oncological treatment is not known.

### The complexity of sleep disturbance and inflammation in cancer patients

Diagnosing cancer-associated inflammation and sleep disruption is complicated by the high incidence of low-grade inflammation and sleep disruption in the elderly population to which cancer patients often belong, even in the absence of malignancy (da Silva et al., 2016; Abd El-Kader and Al-Jiffri, 2019). Other factors that are often co-existing with malignant disease such as menopausal symptoms and circadian rhythm disturbances related to for example steroid treatments could also lead to sleep disturbances and changes in systemic inflammatory profiles in cancer patients (Fox et al., 2020). Some malignancies such as CNS cancers may also disrupt the central circadian organizers (located in the suprachiasmatic nucleus) or sleep organizers in other areas of the brain, which further adds to the risk of developing sleep disruptions in this patient group (Gapstur et al., 2009), Furthermore, toxicities linked to oncological treatments including off-target tissue damage, bone marrow suppression, fatigue, anxiety, pain, and depression to mention a few, are associated with increased risk of *de novo* or deterioration of existing sleep disturbances (Oliva et al., 2019), and an upregulation of pro-inflammatory cytokines (Mills et al., 2008; Wang et al., 2010), which in both cases could persist long after the conclusion of the treatment cycle. As these cytokines might trigger loss of REM sleep (Irwin, 2015; Irwin and Opp, 2017), cancer patients that experience toxic side-effects from such oncological treatment may suffer (further) reduced sleep quality and therefore sleep disruption-associated treatment resistance, progression, reduced quality of life (Oliva et al., 2014) and survival (Innominato et al., 2012; Balachandran et al., 2021). Cancer patients, however, exhibit highly diverse toxic phenotypes and to varying degrees (Oliva et al., 2017), and it is not currently possible to predict which patients will suffer from treatment-associated sleep disruption, or how this can be prevented. Combined, characterizing, and understanding the landscape of sleep disruption and its effect on systemic inflammation in cancer patients undergoing oncological treatment is highly challenging and complex, which complicates the understanding the underlying etiology and prevent the development of effective diagnostic tools.

Here we sought to develop a general view on how oncological therapies may affect sleep and circulating levels of inflammatory and various other plasma proteins across different malignancies and therapies. We further aimed to identify specific biomarkers, or biomarker families, coupled to treatment-associated sleep disruption.

## Materials and methods

### Participants

Ninety patients undergoing systemic oncological treatment for different types of cancer: gastrointestinal cancer, urothelial cancer, breast cancer, brain tumor and tonsillar cancer were included in this study at the Oncology clinic, Ryhov County Hospital, Jönköping, Sweden between 2017 and 2018. The inclusion criteria were patients planned to receive adjuvant or palliative treatment, having an ECOG performance status of 0 or 1, and having sufficient understanding of the Swedish language to understand the information given. The patients were informed about the study orally and on paper by an oncology nurse and were given 30 min or until the next appointment to consider their participation.

The included patients answered the 12-point MOS sleep scale and provided a blood sample (10 ml) both before starting treatment and 3 months after treatment started. Other demographic data were also obtained through a structured anamnesis.

Ethical permission was obtained from the Regional Ethical Review Board in Linköping, Sweden (Dnr 2016/379-31).

### Medical Outcomes Study sleep scale

The MOS – Sleep Scale is a commonly used instrument for subjective evaluation of sleep disturbances in patients with chronic illness or malignant diseases (Naughton et al., 2002; Manas et al., 2011; Hsiao et al., 2013; Sharp et al., 2013). The MOS sleep scale is based on 12 items and takes no more than 5 min to complete. It measures important origins and parameters of sleep, which are significant for cancer patients as well as other patient groups. It includes questions related to sleep initiation, maintenance, adequacy, somnolence, and respiratory impairments (Allen et al., 2009). In this study, the answers to each of the 12 questions were aggregated to a single “aggregated MOS score” by adding the scores given to questions 1, 3b, and 3j (answered on a 1–5 Likert scale where 5 indicate the worst possible sleep quality) to the inverted scores (i.e., six minus the score value) given to questions 3a and 3c-i (also answered on a 1–5 Likert scale where 5 indicate the best possible sleep quality). A value of eight was subtracted from the answer to question 2 (the average number of hours slept per night), and negative values were divided by –1 to result in the (positive) number of additional or fewer hours of sleep compared what is recommended, and this value was added to the other scores. The resulting aggregated MOS score had a minimum value of eleven and a maximum score of 71.

### Laboratory assessments for serum biomarkers

Levels of plasma CRP were analyzed using a high-sensitivity CRP test based on the Advia 1800 instrument and reagents from Siemens (Siemens Healthcare, Erlangen, Germany) allowing detection of CRP levels in the range of 0.16–10 mg/L. When higher levels than 10 mg/L were detected, a standard CRP test with a range of 4–300 mg/L was done on the same instrument and with the same reagents.

Analyses of plasma biomarkers was first done on 17 patients with established good (*n* = 9) or poor (*n* = 8) sleep quality respectively, using Human Cytokine/Chemokine/Growth Factor 45-Plex ProcartaPlex Panel 1 (Thermo Fisher Scientific, Austria). Out of this panel of 45 biomarkers, 17 (BDNF, Eotaxin, GM-CSF, GRO-a, HGF, IFN-g, IL-10, IL-12p40, IL-2, IL-5, IL-6, IL-7, LIF, MCP-1, PIGF-1, RANTES, and TNF-a) were selected based statistical significance of *p* < 0.10 when comparing groups of “good” versus “poor” sleeping cancer patients at baseline and when comparing patients before and after 3 months of oncological treatment. Analyses of the 17 biomarkes were done with a custom procartaplex 17-plex (Thermo Fisher Scientific, Austria) using multiplex fluorochrome technique, (Luminex xMAP™ Technology, Austin, TX, USA) according to the manufacture’s recommendations. A Bio-Plex 200 system with Bio-Plex Manager Software 5.0 were used to collect the fluorescence intensities. A 9-standard concentration set (included in the kit) were used to generate a standard curve for the calculation of the analyte concentration.

The 17 biomarkers were grouped into one of 5 biomarker families based on consensus from the collected scientific literature of their known or best described function. As such, TNF-a, IFN-g, IL-6, IL-2, IL-12, and GM-CSF were classified as “pro-inflammatory cytokines,” IL-10 and LIF were classified as “anti-inflammatory cytokines,” BDNF, PLGF, and HGF were classified as “growth factors,” Eotaxin, Rantes, MCP-1, and GRO were classified as “chemokines” and IL-5, IL-7, and CRP were classified as “modulatory and other” biomarkers. This classification should however not be seen as an exclusive function of a given factor within the family.

Aggregated plasma values for an entire biomarker family was generated by normalizing the concentrations for each biomarker and patient against the average of the patient population (e.g., dividing the concentration of for example BDNF for a given patient by the average for the entire population), followed by averaging such normalized levels of all biomarkers within the family (e.g., averaging normalized levels of BDNF, PLGF, and HGF from a given patient to derive the aggregated plasma value for the “growth factors” biomarker family for that patient).

### Statistics

**FIGURE 1:**
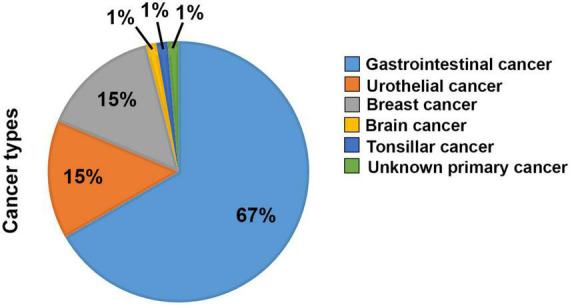
Overview of the diagnoses of the patients included in the study. Percentages of the 90 patients included having either a Gastrointestinal (60), Urothelial (13), Breast (14), Brain (1), Tonsilar (1), or Unknown Primary (1) cancer diagnosis.

**FIGURE 2:**
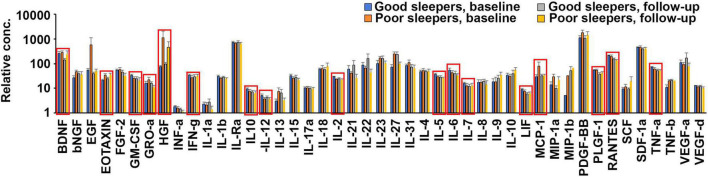
Poor and/or deteriorated sleep quality during oncological treatment is coupled to changes in plasma levels of 17 cytokines. Quantification of the average levels of 45 cytokines in the plasma of patients reporting good (aggregated MOS score < 24, *n* = 9) and poor (aggregated MOS score > 25, *n* = 8) sleep at baseline or follow-up, after 3 months of oncological treatment. Significant (*p* < 0.1) differences were observed between good and poor sleepers at baseline for Eotaxin, IFN-g, IL12, IL2, IL5, and MCP-1, between good and poor sleepers at follow-up for GRO-a and IL6 and for those exhibiting good sleep at baseline that deteriorated to poor sleep at follow-up for BDNF, GM-CSF, HGF, IL10, IL5, IL7, LIF, PlGF, RANTES, and TNF-a. These 17 biomarkers are indicated with red boxes in the graph.

**FIGURE 3:**
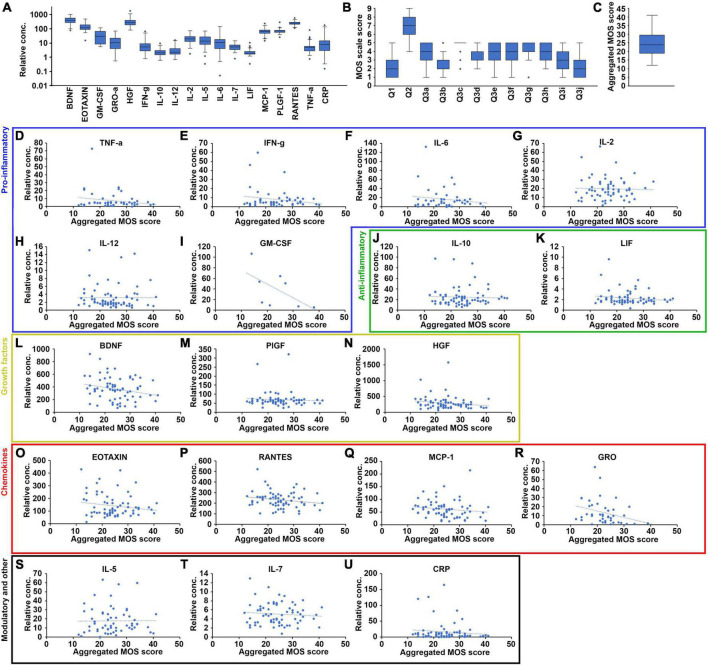
Correlation between cytokine biomarker plasma levels and sleep quality at baseline for some but not all of the 17 selected cytokines. **(A)** Quantification of the plasma levels of the 17 biomarkers for the fully evaluated patients at baseline, shown as a box-plot with the median values represented as a black lines, and the 95% confidence intervals contained within the error bars (*n* = 71). **(B)** Quantification of the range of scores given to each of the 12 questions included in the MOS sleep scale for the fully evaluated patients at baseline, shown as a box-plot with the median values represented as black lines and the 95% confidence intervals contained within the error bars (*n* = 71). **(C)** The range of aggregated MOS scores calculated form the answers to the 12 individual questions of the MOS sleep scale for the fully evaluated patients at baseline, shown as a box-plot with the median value represented as a black line and the 95% confidence interval contained within the error bars (*n* = 71). Plasma levels of each of the 17 selected biomarkers plotted against the aggregated MOS score of the same patient, for all fully evaluated patients at baseline. The biomarkers were organized into functional groups including pro-inflammatory (**D–I**, blue box), anti-inflammatory (**J,K**, green box), growth factors (**L–N**, yellow box), chemokines (**O–R**, red box), and immune-modulatory and other (**S–U**, black box) biomarkers. *N* = 71, blue dashed lines indicate linear regression curves for the sample population.

**FIGURE 4:**
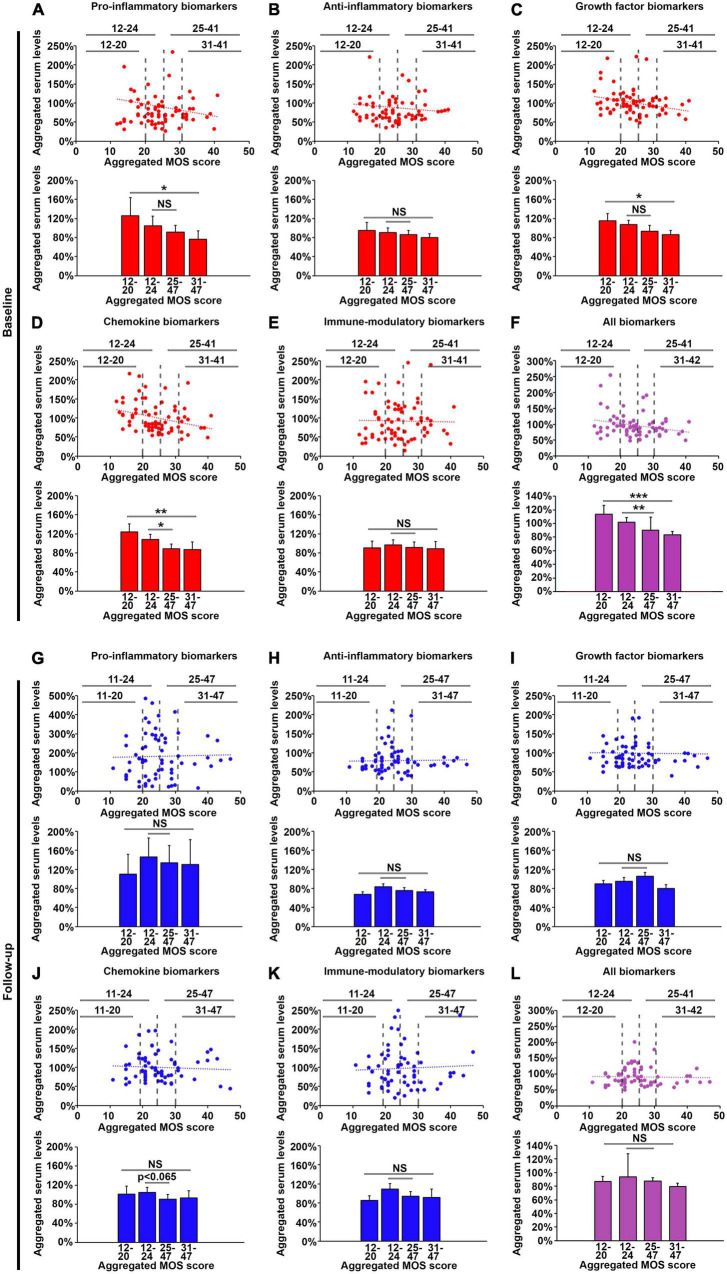
Pro-inflammatory, growth factor and chemokine biomarkers in the plasma are lowered in patients exhibiting poor sleep at baseline, but not at follow-up. Average levels of normalized plasma concentrations for biomarkers in each of the five categories pro-inflammatory biomarkers **(A,G)**, anti-inflammatory biomarkers **(B,H)**, growth factor biomarkers **(C,I)**, cytokine biomarkers **(D,J)** and immune-modulatory biomarkers **(E,K)**, as well as for all 17 biomarkers (purple graphs, **F,L**) at baseline (red graphs, **A–F**) and follow-up (blue graphs, **G–L**). The top graphs show the normalized, averaged levels of these biomarkers plotted against the aggregated MOS score for each patient, and the lower histographs show the averaged levels of these biomarkers for patients within four sleep quality groups: very good sleep (aggregated MOS score < 20), good sleep (aggregated MOS score < 24), poor sleep (aggregated MOS score > 25), and very poor sleep (aggregated MOS score > 31). *N* = 71. *p < 0.05, **p < 0.01, ***p < 0.001, NS: non-significant.

**FIGURE 5:**
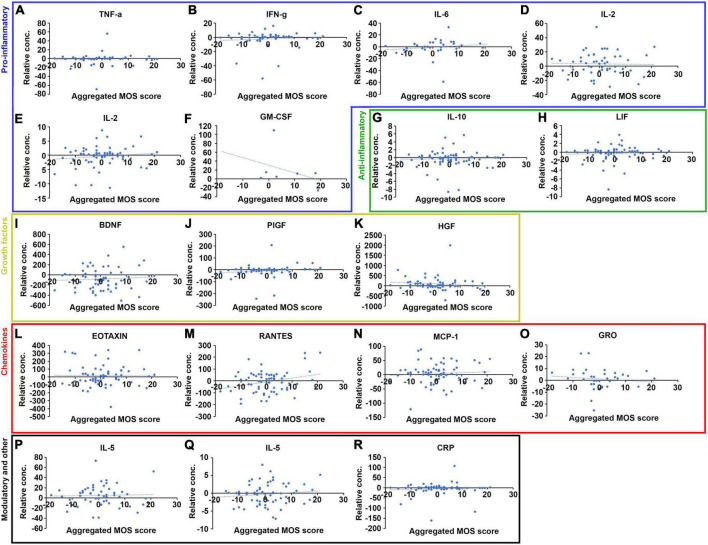
Correlation between changes in cytokine biomarker plasma levels and changes in sleep quality between baseline and follow-up for a few of the 17 selected cytokines. Changes in plasma levels plotted against changes in aggregated MOS score, calculated as the plasma level or MOS score at follow-up minus the plasma level/MOS score at baseline, for each of the 17 selected cytokines and organized into functional groups including pro-inflammatory (**A–F**, blue box), anti-inflammatory (**G–H**, green box), growth factors (**I–K**, yellow box), chemokines (**L–O**, red box) and immune-modulatory and other (**P–R**, black box) biomarkers. *N* = 71, blue dashed lines indicate linear regression curves for the sample population.

**FIGURE 6:**
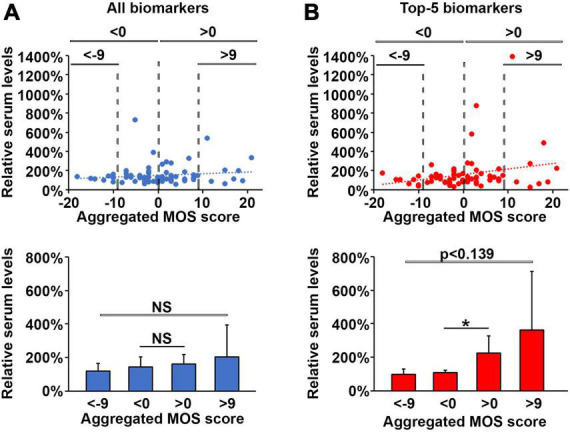
An increase in pro-inflammatory biomarkers is associated with loss of sleep quality during oncological treatment. Normalized changes in plasma levels of all 17 selected cytokines **(A)** or pro-inflammatory cytokines **(B)** with values lower than 100% indicating a reduction in cytokine levels at follow-up compared to baseline and plotted against changes in aggregated MOS scores with negative values indicating improved sleep quality whereas positive values indicate deteriorated sleep quality at follow-up compared to baseline. The lower histographs show the average normalized values for these cytokines among patients with very good sleep (aggregated MOS score < 20), good sleep (aggregated MOS score < 24), poor sleep (aggregated MOS score > 25), and very poor sleep (aggregated MOS score > 31). *N* = 71. *p < 0.05, NS, non-significant.

**FIGURE 7:**
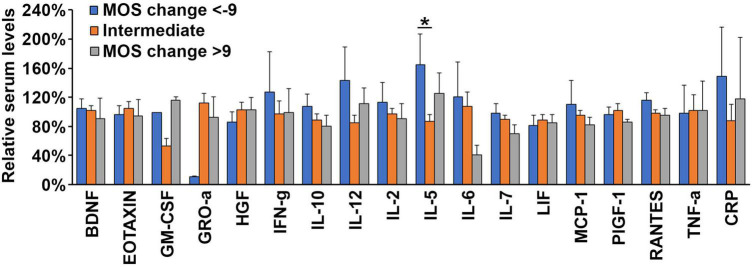
High serum levels of IL5 is associated with sleep improvement during oncological therapy. Average levels of normalized plasma concentrations of each of the 17 biomarkers selected for this study and CRP within groups of patients exhibiting a large gain in sleep quality (MOS change < -9, blue bars), an intermediate gain or loss of sleep quality (MOS change < ±9, orange bars) and a large loss of sleep quality (MOS change > 9, gray bars) after a round of oncological therapy. *N* = 71. *p < 0.05.

All data was considered to not significantly deviate from binomial distribution and is therefore presented as means ± standard error (Figures 1, 3, 5, 6), alternatively as means with 95% confidence intervals shown as box-plots (Figure 2) or as swarm plots with linear inserted regression graphs (Figures 2, 4). Where means are presented in Figures 2, 3A–C these are means of the entire population of 71 patients. Means presented in Figures 4, 6 represent the means of the patients within the given sleep quality group, where the individual values are shown in the swam plots directly above the histograms. Means in Figure 7 represent values from 8, 55, and 8 patients in the < -9, intermediate and > 9 groups respectively. Changes in biomarker levels or MOS scores in a patient were derived by subtracting the values at baseline from those measured at follow-up. Differences between two groups were evaluated using students *t*-test as shown using red boxes in Figure 2, black lines connecting the two groups being compared and coupled to statistical indicators (NS: non-significant, *p < 0.05, ^**^p < 0.01 and ^***^p < 0.001), alternatively *p*-values, placed directly above the lines in Figures 4, 6, 7. Correlation lines shown in swam plots of Figures 3–6 illustrate linear regressions, the significance of which could not be uniformly calculated due to many missing values for a few of the biomarkers, and are therefore omitted.

## Results

A total of 90 patients diagnosed with gastrointestinal (67%), urothelial (15%), breast (15%), brain (1%), tonsillar (1%), or unknown primary (1%) cancer were recruited to the study (Figure 1). Among these, nineteen patients failed to provide the requested follow-up samples or withdrew their consent without disclosing the reason, and could therefore not be fully analyzed. Medical treatment consisted mainly of high-dose cytostatic and/or cytotoxic drugs but varied depending on various clinical factors in accordance with current treatment guidelines. Approximately 2/3 were male at a median age of 68 and 1/3 were female at a median age of 61 years. Additional demographic, diagnostic, and co-morbidity-data from the study population is presented in Table 1.

**TABLE 1:** Demographic data.

Parameter	*n* = (%)
Age (min–max)	31–82
Female	28 (37)
Male	47 (63)
ECOG Performance Status*	
0	46 (63)
1	29 (37)
Alcohol consumption	
No	23 (30)
Yes	52 (70)
Nausea experience before treatment	
Pregnancy nausea (only female)	8 (11)
Travel nausea	7 (9)
No nausea at all	60 (80)
Occupation	
Retired	49 (65)
Worker	25 (33)
Unemployed	1 (2)
Civil State	
Married/Partner	64 (85)
Single	10 (13)
Widower	1 (2)
Smoking	
No	65 (87)
Yes	10 (13)
BMI	
Min	19
Max	43
Average	26
Tumor classification	
Gastrointestinal cancer (colorectal, pancreas, liver, esophageal)	50
Urotelial cancer (Bladder, prostate)	11
Breast cancer	11
Brain tumor	1
Tonsillar cancer	1
Cancer of unknown primary	1
Comorbidity	
Hypertension	9
Diabetes	8
Hypothyreosis	1
Hypercholesterolemia	1
Rheumatoid arthritis	1
Chronic obstructive pulmonary disease including Asthma	4
Depression	1

*ECOG, Eastern Cooperative Oncology Group.

Plasma levels of 45 biomarkers were analyzed from nine patients who reported high sleep quality (i.e., low aggregated MOS scores) and eight who reported poor sleep quality (i.e., high aggregated MOS scores). We found that Eotaxin and MCP-1 were significantly upregulated, and IFN-g, IL-12, IL-2, and IL-5 were significantly downregulated (*p* < 0.1, using students *t*-test) in poor sleepers versus good sleepers at baseline. Compared to baseline levels we also found that HGF was significantly upregulated and BDNF, GM-CSF, IL-10, IL-5, IL-7, LIF, PlGF-1, RANTES, and TNF-a were significantly downregulated (*p* < 0.1, using students *t*-test) at follow-up in good sleepers and IL-6 and GRO-a were significantly downregulated (*p* < 0.1, using students *t*-test) in poor sleepers (Figure 2). We therefore selected these 17 proteins for further analysis in the entire patient cohort.

The 17 biomarkers exhibited great variation among the patients at baseline (Figure 3A). While the plasma levels measured in most patients were within (broad) 95% confidence intervals, a few patients exhibited levels significantly above or below these “reference” levels. Similarly, the scores given to the 12 MOS scale questions also varied greatly at baseline (Figure 3B) and some of the MOS scale scores exhibited dramatically increased or reduced values for a few of the patients compared to the 95% confidence intervals. This was particularly clear for the answers to question 3c related to having shortness of breath or headache when waking up in the morning. All patients, however, fit within the 95% confidence interval for the aggregated MOS score (Figure 3C). Furthermore, the aggregated MOS score did not differ significantly among different patient subgroups including patients with different cancer diagnoses, age at diagnoses or treatment regimen (Supplemental Figure 1), suggesting that the this is a more robust measure for sleep disruption in cancer patients compared to any of the questions in isolation.

We next investigated the relationship between the levels of the 17 investigated biomarkers and sleep quality as measured by the aggregated MOS score (Figures 3D–U). Plasma concentrations of these 17 biomarkers or CRP did not exhibit any obvious correlation to the sleep quality measured by the aggregated MOS score within this cohort, suggesting that both the regulation of these biomarkers as well as sleep physiology is complex in cancer patients and that sleep disruption cannot be accurately predicted by any single biomarker. Grouping the biomarkers into five families, and analyzing changes in aggregated plasma levels for entire families between different sleep quality patient groups using student *t*-test (Figure 4), we found that the factors within the pro-inflammatory, growth factor and chemokine families were significantly reduced (*p* < 0.05, *p* < 0.05, and *p* < 0.01, respectively) in the lowest sleep quality (aggregated MOS score > 30) compared to the highest sleep quality (aggregated MOS score < 21) groups. Chemokine biomarkers, furthermore, were significantly reduced (*p* < 0.05) in the entire population of poor sleepers (aggregated MOS score > 24) compared to good sleepers (aggregated MOS score < 25, Figures 4A–E). Aggregating all 17 biomarkers and CRP levels into a single value for each patient demonstrated that this panel of biomarkers were robustly reduced both for all poor sleepers (aggregated MOS score > 25) compared to good sleepers (*p* < 0.01) but even more so among those having the worst (aggregated MOS score > 31) compared to the best (aggregated MOS score < 20, *p* < 0.001, Figure 4F) sleep quality.

We next investigated if the biomarker levels in plasma were affected by the 3 months oncological treatment regimen. Interestingly, patients who slept well (aggregated MOS score < 24) after the medical treatment period now exhibited lower aggregated levels of growth factor and chemokine family biomarkers, as well as the global aggregation of all 17 biomarkers and CRP compared to baseline (Figures 4I,J,L). On the other hand, pro-inflammatory biomarkers were elevated among poor sleepers at follow-up compared to baseline (Figure 4G) leading to none of the biomarker families being predictive of sleep quality in patients after 3 months of oncological treatment (*p* > 0.05 using students *t*-test, Figures 4G–L). These results highlight the complexity and dynamics of circulating biomarkers of sleep disruption during oncological treatment in cancer patients and suggest that commonly studied biomarkers such as sub-pathologically elevated CRP (detected by a high sensitivity test) or pro-inflammatory cytokines are not predictive for evaluating sleep disruption in cancer patients undergoing treatment.

To understand whether the oncological treatment caused the changes in biomarker levels, alternatively to what extent this may be coupled to a concurrent improvement or reduction in sleep quality, we investigated how the aggregated MOS scale values and biomarker plasma levels changed from baseline to follow-up for each individual patient. Whereas some biomarkers including TNF-a, IFN-g, PlGF, HGF, and CRP did not change for almost any of the patients (Figures 5A,B,J,K,R), many biomarkers were found to either increase or decrease dramatically in individual patients (Figures 5C–I,L–Q). The aggregated changes in plasma levels for the biomarker families demonstrated that all 17 biomarkers combined did not correlate to a improvement or deterioration in sleep quality (Figure 6A). However, the levels of pro-inflammatory biomarkers increased significantly (*p* > 0.05 analyzed using students *t*-test) in patients whose sleep quality deteriorated during treatment (change in aggregated MOS score > 0) compared to those whose sleep quality improved (change in aggregated MOS score < 0, Figure 6B). Taken together, these findings suggest that analyzing at the plasma levels of proinflammatory cytokines only after an oncological treatment cycle will not allow identification of patients with good or poor sleep quality (Figure 4G) but measuring the change in the levels of these factors, however, might be highly indicative of experienced improvement in or deterioration of sleep quality.

Next, we hypothesized that the expression level of some of the investigated plasma proteins at baseline might be associated with increased risk of or protection from losing sleep quality during an oncological treatment cycle. However, only one cytokine, IL-5, exhibited significantly higher plasma concentrations (*p* < 0.05 using students *t*-test) at baseline in patients that dramatically gained sleep quality (change in aggregated MOS score < -9) during the 3 months treatment period (Figure 7). As none of the biomarkers were changed at baseline in the patients whose sleep quality deteriorated during treatment, it was not possible to establish a prognostic principle for predicting this adverse effect. We do, however, suggest that further studies investigate the potential role of IL-5 in protecting against oncological treatment-induced sleep deterioration.

## Discussion

In this study we have evaluated the diagnostic value of 45 circulating biomarkers associated with immune function for identifying subjective sleep disruption in cancer patients. First, to improve and standardize the evaluation of sleep quality and -disruption in cancer patients, we developed a new framework for analysis of a popular sleep-instrument, the MOS sleep scale by a new aggregated MOS score, which is the first score based on this instrument that includes all of the 12 questions of the scale. This aggregated MOS score represents a single value related to the sleep quality of the patient which we propose allows a stronger, more robust and intuitive use of the MOS sleep scale compared to the various sub-scale scores that have been used in the past (Agrafiotis et al., 2022). We found that, at baseline, the mean of this aggregated MOS score was 24,4 and that patients with a score of 25 or higher (indicating poor sleep) exhibited significantly lower levels of 17 among the original 45 biomarkers analyzed, compared to those having an aggregated MOS score of 24 or lower (indicating good sleep). The differences were, however, higher for patients with more severe sleep disruption (aggregated MOS score of 31 or higher) compared to those with an aggregated MOS score of 20 or lower. Growth factor-, chemokine- and pro-inflammatory biomarkers were more strongly suppressed in patients with severe sleep disruption compared to anti-inflammatory and immune-modulatory biomarkers. This was, however, only seen at baseline, prior to initiation of oncological therapy. At the first follow-up examination, after 3 months of treatment, the correlation between sleep quality and plasma levels of all biomarkers analyzed were completely abrogated. In the case of the pro-inflammatory biomarkers, this abrogation was mainly due to a dramatic increase in the serum levels of these markers in patients reporting poor sleep quality, suggesting that therapy-induced inflammation is unabated if sleep quality is impaired. Indeed, plasma concentrations of pro-inflammatory biomarkers were found to increase several fold during oncological therapy but only in patients whose sleep quality concurrently deteriorated during this period. These findings are in line with previous studies showing that pro-inflammatory biomarkers such as IL-1b, IL-2, IL-6, IL-12, and TNF-a increase in cancer patients following a period of sleep disruption (Mantovani et al., 2008; Jensen et al., 2021; Tucker et al., 2021). Surprisingly, patients reporting improved sleep quality during oncological therapy exhibited reduced plasma levels of pro-inflammatory cytokines clearly demonstrating that sleep disruption rather than cytostatic or cytotoxic therapy is driving the increased systemic inflammation observed in these patients.

Previous studies aiming to identify biomarkers of sleep disruption in cancer patients have been designed to investigate relatively homogeneous patient cohorts. In such studies, survivors of childhood ALL exhibiting poor sleep were found to have elevated plasma levels of IL-6, IL1b and CRP (Cheung et al., 2017), and elevated IL-6 levels were correlated to poor sleep in non-small cell lung cancer patients receiving chemoradiotherapy (Wang et al., 2010). Tucker et al found that IL-2, IL-1b, and IL-6 were upregulated in cancer survivors exhibiting reduced sleep duration based on actigraph measurements, but not in patients with self-reported sleep disruption where instead IL-10 were found to be upregulated (Tucker et al., 2021). Here we took a different approach by keeping the inclusion criteria broad: All cancer patients with performance status 0 or 1, scheduled for oncological therapy were invited to participate regardless of age, gender, cancer type, tumor stage, type of planned oncological therapy, pre-treatment status, prior surgery, etc. Looking specifically at adjuvant versus palliative therapy, type of drugs selected for treatment and tumor types, we saw that these parameters did not influence the plasma levels of the biomarkers examined in this study. The heterogeneity of this cohort may, nevertheless, contribute to why no single biomarker was found to significantly correlate with sleep disruption in this study. As such, while other studies have found that IL-6, IL-1b, and CRP is significantly up-regulated in specific groups of cancer patients exhibiting poor sleep (Wang et al., 2010; Cheung et al., 2017), this/these biomarker(s) did not correlate significantly with sleep quality in our broader cohort. Taking an OMICs-like approach, we instead identified functional panels of biomarkers that combined were robustly correlated to sleep disruption even in our broad patient cohort. In particular the pro-inflammatory markers TNF-a, IFN-g, IL-6, IL-12, IL-2, and GM-CSF constituted one such pro-inflammatory panel that could predict both sleep disruption at baseline (in patients expressing below-average levels of these biomarkers) as well as deteriorating sleep quality during treatment. Some of the factors analyzed in this study may have several, context-dependent functions. GM-CSF, for example, in addition to being a pro-inflammatory cytokine, also has both chemokine and growth factor properties as this factor is used to mobilize and expand hematopoietic stem cells from the bone-marrow in cancer patients during chemotherapy (Siena et al., 1989). The chosen classification for the factors analyzed in this study therefore reflects how these factors are described by the bulk of the literature. Some of the factors found in the pro-inflammatory cytokines panel are well known biomarkers of circadian and sleep disruption (TNF-a and IL-6, see above), whereas the others have not previously been coupled to sleep disruption in cancer patients (e.g., GM-CSF).

A cancer diagnosis may, by itself, cause sleep disruptions as the incidence of sleep disruption is on average three times higher among cancer patients compared to the general population, with breast cancer patients being at particularly high risk (Palesh et al., 2010; Yennurajalingam et al., 2018). In our study we found that the subsequent oncological treatment did not further affect the extent of sleep disruption, within the study population in general, with the possible exception of patients treated with taxanes, which seemed to further deteriorate their sleep quality compared to other types of treatment. Furthermore, sleep disruption may persist long into survivorship, even months or years after completed treatment (Otte et al., 2009). While individual patients may improve or further disrupt their sleep quality following treatment, these findings strengthen the view that medical therapy does not impact the sleep quality of cancer patients in general.

A weakness of this study is the relatively small size of the study cohort, especially considering the broad inclusion criteria. As a likely consequence of this, we did not find single factors that correlated with poor sleep quality at baseline or a loss of sleep quality during oncological therapy. The broad inclusion criteria could, however, also be seen as a strength as our finding that (small) panels of inflammatory biomarkers exhibit robust correlation with sleep disruption across diagnoses and treatment regimens, are likely more generalizable within cancer care. While we included patients with a variety of malignancies, it should be noted that colorectal cancer patients were somewhat overrepresented (47%) in this patient cohort. Indeed, among the cancers with high incidence in Sweden, colorectal cancer is commonly treated aggressively using chemotherapeutic agents, which is not as common in for example prostate or breast cancer patients, which could explain why such patients were favored for inclusion in this study. Furthermore, both the oncology infrastructure at the study site and differences in the motivation and workload among patient-recruiting nurses within different specialities may also play a role in determining the distribution of patients across different diagnoses in this study. Other studies looking at sleep disruption in cancer patients and correlating this to effects of treatments have similar cohort sizes to that of the present study (Innominato et al., 2012; Jensen et al., 2021), but are focusing on more defined patient populations. Innominato et al. (2012), for example, studied circadian disruption using Actigraph accelerometers worn on the wrist for 3 days and found that sleep disruption in 77 patients with metastatic colorectal cancer undergoing medical treatment with 5-FU, Oxaliplatin and/or Leucovorin had significantly shortened survival compared to those with non-disrupted sleep (Innominato et al., 2012). Similarly, Cash et al reported significantly shorter overall survival among patients exhibiting disrupted activity/rest rhythms in a cohort of 55 head and neck cancer patients undergoing chemoradiation therapy (Cash et al., 2018). None of these studies, however, investigated circulating biomarkers or inflammatory profiles in the patients. In a meta-analysis on sleep disruption in patients with different types of CNS cancer, a particularly complex patient group in this context as their sleep physiology may also be directly affected by their disease or treatment, 24 of the 25 studies included between 1 and 115 patients (20 patients per study on average) (Gapstur et al., 2009). While our study should still be seen as a pilot study, it does in this context have a relatively high power. The potential of the suggested panel of six pro-inflammatory biomarkers as a tool for diagnosing sleep disruption in cancer patients both before and after oncological treatment initiation, however, needs to be validated in larger studies in the future.

## Conclusion

In conclusion, in this study we found that while 17 of 45 small plasma proteins studied were individually de-regulated in poor compared to good sleepers in a small sub-cohort of 17 cancer patients, this could not be reproduced in a larger cohort of 71 patients. However, combining factors with a similar mode of action into panels of biomarkers and deriving an aggregated measure of how such panels were deregulated allowed identification of a panel of six pro-inflammatory biomarkers exhibiting high accuracy and robustness for predicting sleep disruption following a 3-month oncological therapy cycle in the complete, broad cohort of cancer patients independent of diagnosis, age or therapy. This is the first-time plasma protein biomarkers have found to correlate with sleep disruption during treatment in such a broad cohort of cancer patients. Furthermore, changes in these pro-inflammatory biomarkers could in this study be un-coupled from therapy-induced inflammation and were found to be completely dependent on changes in sleep quality after 3 months of treatment. This interesting finding warrants further mechanistic studies in the future.

## Data availability statement

The raw data supporting the conclusions of this article will be made available by the authors, without undue reservation.

## Ethics statement

The studies involving human participants were reviewed and approved by the Ethical Review Board in Linköping, Sweden (2016/379-31). The patients/participants provided their written informed consent to participate in this study.

## Author contributions

DO, B-ÅA, FL, and LJ designed the study, analyzed data, and wrote the manuscript. DO conducted clinical examinations, recruited patients, and performed follow-up analysis of patient journals. B-ÅA performed laboratory experiments and preliminary data analysis. All authors contributed to the article and approved the submitted version.
