# Supplementary figures and images for: Opposing inflammatory biomarker responses to sleep disruption in cancer patients before and during oncological therapy
Source: Front Neurosci. 2022 Sep 21;16:945784. doi: 10.3389/fnins.2022.945784 (PMC9534604; doi:10.3389/fnins.2022.945784)

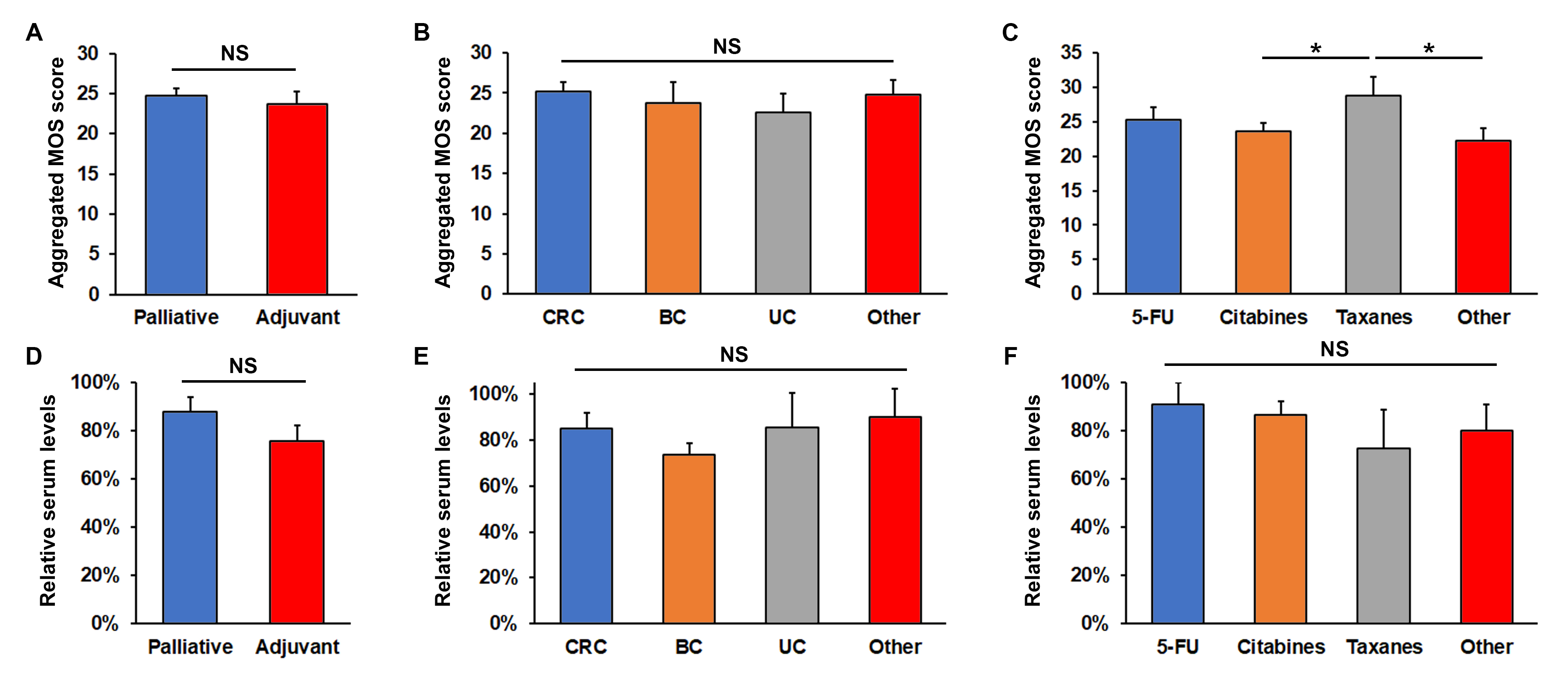

Supplement: Supplementary file 1 [file Image_1.JPEG]
